# Supplementary material for: Divergence in female damselfly sensory structures is consistent with a species recognition function but shows no evidence of reproductive character displacement
Source: Ecol Evol. 2018 Nov 20;8(23):12101–14. doi: 10.1002/ece3.4669 (PMC6303706; doi:10.1002/ece3.4669)
Supplement: Supplementary file 1 [file ECE3-8-12101-s001.docx]

**Table S1.** Statistical comparison of sensilla traits among sympatric, locally allopatric, and fully allopatric *E. carunculatum* populations.

|  | Mean + s.e.m. | | |  |  |
| --- | --- | --- | --- | --- | --- |
| Trait | Sympatry S  (N = 1) | Local allopatry  (N = 2) | Allopatry  (N = 3) | χ^2^_2_^†^ | *P* |
| Sensilla number | 25.8 | 26.9 + 2.6 | 29.0 + 0.4 | 2.79 | 0.25 |
| Proportion plate containing sensilla | 0.21 | 0.23 + 0.30 | 0.21 + 0.23 | 0.04 | 0.98 |
| Sensilla density (sensilla mm^-2^) | 15.1 | 17.3 + 0.2 | 15.9 + 0.8 | 2.14 | 0.34 |
| Mean distance (µm) between sensilla pairs | 3.27 | 2.85 + 0.02 | 2.91 + 0.32 | 0.86 | 0.65 |

* N refers to the number of populations analyzed.

† χ^2^_2_ refers to the Kruskal-Wallis chi-squared value with 2 degrees of freedom.


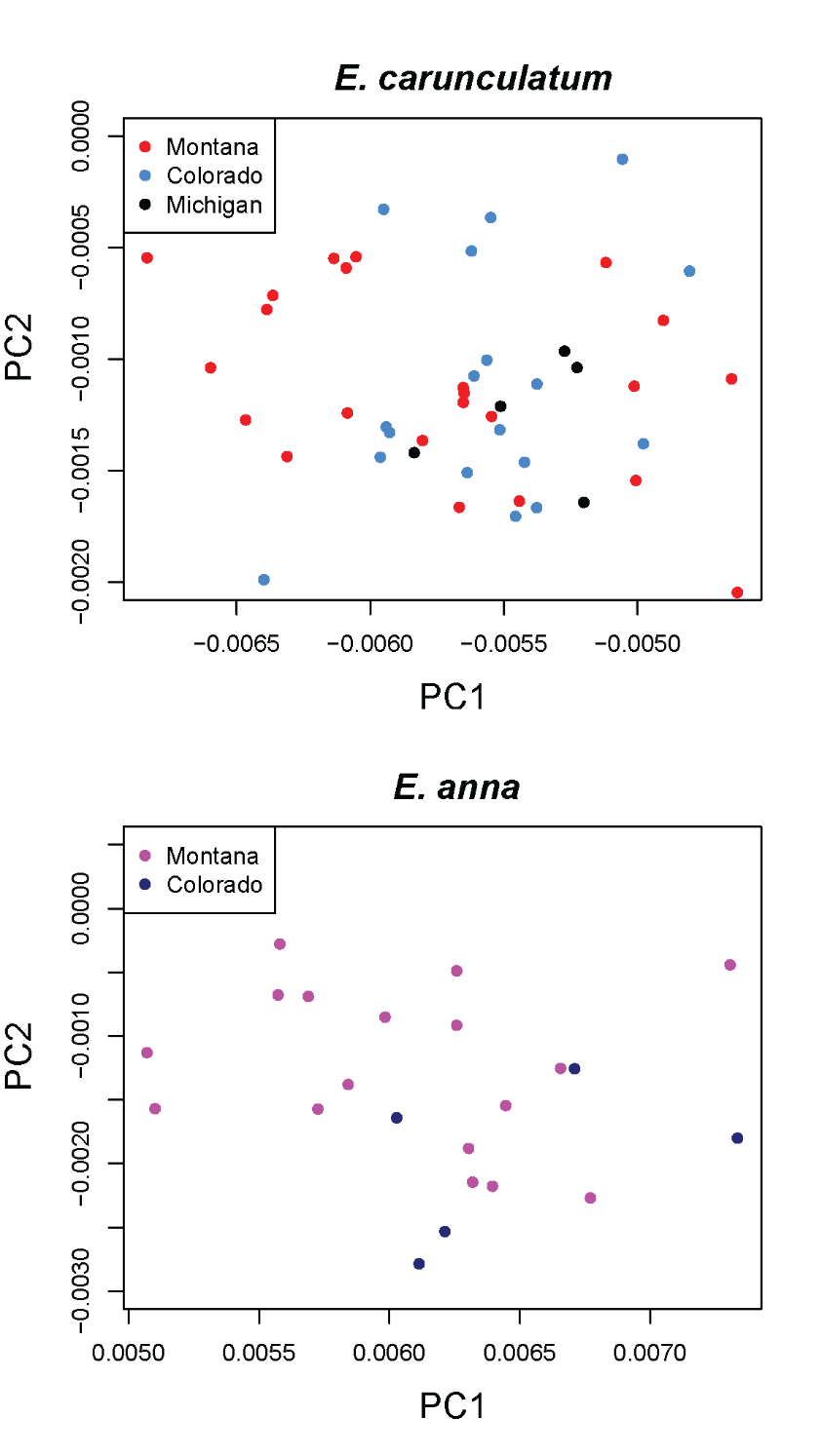


**Figure S1.** Results of principal component analysis of spherical harmonic coefficients describing 3D shapes of male cerci demonstrate that within each species, cercus morphology is similar among geographically distinct populations. Methods and Montana data are published in Barnard et. al (2017).
